# Whole-genome sequencing of *Bacillus subtilis* KnB1, a biological control agent isolated from bean rhizosphere in Thailand

**DOI:** 10.1128/mra.00396-26

**Published:** 2026-06-15

**Authors:** Nutjarin Haewou, Choosak Khaengraeng, Thanikan Niamtaeng, Tiyakhon Chatnaparat

**Affiliations:** 1 Department of Plant Pathology, Faculty of Agriculture, Kasetsart University 54775, Bangkok, Thailand; Rochester Institute of Technology, Rochester, New York, USA

**Keywords:** PGPR, beneficial bacteria, biological control agent, *Bacillus *biofertilizers

## Abstract

*Bacillus subtilis* strain KnB1 isolated from the rhizosphere of *Vigna unguiculata* (L.) Walp. exhibited biocontrol activity against diverse plant pathogens. We assembled a high-quality genome using Oxford Nanopore and Illumina sequencing. The KnB1 genome comprises a 4,084,082 bp circular chromosome with 43.81% guanine-cytosine (GC) content, providing valuable insights into its biocontrol potential.

## ANNOUNCEMENT

Strain KnB1 was isolated in June 2016 from rhizosphere soil tightly adhering to bean roots in Tha Muang District, Kanchanaburi Province, Thailand. One gram of soil was suspended in 9 mL of distilled water, serially diluted (10⁻¹–10⁻⁴) in phosphate-buffered saline (PBS), and plated on nutrient agar (NA). Colonies of KnB1 were white, irregular form, with undulate margins. In this study, the complete genome of strain KnB1 was sequenced due to its broad-spectrum antagonistic activity against various plant pathogens. Furthermore, its secondary metabolite gene clusters and plant interaction-related functional genes were annotated, providing a molecular basis for understanding its antagonistic properties and plant growth-promoting mechanisms.

For genome sequencing, a single colony of strain KnB1 was inoculated into nutrient broth (NB) and cultured in a shaker at 250 rpm and 28°C for 24 h. Cells were harvested by centrifugation at 10,000 × *g* for 5 min. High-quality genomic DNA was extracted using the DNeasy PowerSoil Pro Kit (Qiagen, Germany) following the manufacturer’s instructions. The genome of strain KnB1 was sequenced using Oxford Nanopore Technology (ONT, UK) at the KKU National Phenome Institute (Khon Kaen, Thailand) and the Illumina NovaSeq platform at Macrogen, Inc. (Seoul, Republic of Korea).

For long-read sequencing, ONT libraries were prepared using the Rapid Barcoding Kit 24 V14 (SQK-RBK114.24, ONT) according to the manufacturer’s recommendations and sequenced on an R10.4.1 (FLO-MIN114) flow cell (ONT). ONT basecalling was performed using the Dorado super-accuracy model (dna_r10.4.1_e8.2_400bps_sup@5.0.0).

A total of 22,619 reads, ranging in length from 14,215 to 98,439 bp, were obtained. For short-read sequencing, the Illumina sequencing library of strain KnB1 was prepared using the TruSeq Nano DNA Library Kit (Illumina, San Diego, CA, USA) and sequenced (2 × 150 bp) on the Illumina NovaSeq 6000 platform following the manufacturer’s protocol at Macrogen, Inc. A total of 21,861,034 paired-end reads, corresponding to 3,301,016,134 bp, were obtained.

Reads were processed and assembled using KBase (1), with default parameters. Read quality was assessed using FastQC v0.12.1 and Compute Simple Read Library Stats v2.0.2. Low-quality reads and adapter sequences were trimmed (>Q30) using the JGI RQCFilter pipeline (BBTools v38.22) v0.5.0, Trim Galore v0.6.5dev, and Cutadapt v4.2 (2). Nanopore reads were quality-filtered with Filtlong (Galaxy Version 0.3.1+galaxy0) (3). Hybrid assembly using both short- and long-read data was performed with Unicycler v0.5.1 (4) using default parameters. Assembly using reads from both methods in Unicycler resulted in one contig with an N_50_ value of 4,083,982 bp and Illumina read-based coverage (coverage, ∼809×).

After *de novo* assembly, genome completeness and contamination were evaluated using CheckM v1.1.2 (5), indicating 99.59% completeness and 0% contamination. GTDB-Tk (Galaxy Version 2.6.1+galaxy0) classified strain KnB1 within the *Bacillus subtilis* species cluster, with an average nucleotide identity (ANI) value of 98.73%. Coding sequences were predicted using the National Center for Biotechnology Information (NCBI) Prokaryotic Genome Annotation Pipeline (PGAP) v6.10 (6). The genome was also annotated using antiSMASH v8.0 (7) to identify biosynthetic gene clusters (BGCs).

The genome of *B. subtilis* strain KnB1 consists of a single circular chromosome with a total length of 4,084,082 bp and an average guanine-cytosine (GC) content of 43.81%. The genome contains 4,071 protein-coding sequences (CDSs), 10 ribosomal RNA (rRNA) genes, 86 transfer RNA (tRNA) genes, and 105 pseudogenes (Table 1).

**TABLE 1:** Genome summary statistics of *Bacillus subtilis* KnB1

Genomic feature	Result
Size (bp)	4,084,082 bp
GC content (%)	43.81%
No. of contigs	1
*N*_50_ (bp)	4,083,982
Genes (total)	4,192
CDSs (total)	4,071
Genes (coding)	3,966
Genes (RNA)	121
rRNA	10, 10, 10 (5S, 16S, 23S)
tRNA	86
ncRNAs	5
Pseudo genes (total)	105

Analysis of BGCs of *B. subtilis* KnB1 revealed the presence of nonribosomal peptide synthetase (NRPS) and polyketide synthase (PKS) gene clusters associated with antimicrobial activity. These include gene clusters responsible for the biosynthesis of surfactin, fengycin, bacillaene, bacilysin, bacillibactin, subtilosin, and a candidate cluster for brevicidine, a promising antimicrobial compound. These clusters are involved in the production of secondary metabolites with antifungal and antibacterial properties. In addition, the presence of several genes contributing to plant growth-promoting (PGP) traits was predicted using the RAST Toolkit (RASTtk) (8). Strain KnB1 harbors genes associated with auxin biosynthesis, siderophore production (anthrachelin and bacillibactin), GABA metabolism (*gabR*), and potassium, phosphorus, and nitrogen metabolism, as well as genes involved in the production of volatile organic compounds, including 3-hydroxy-2-butanone (acetoin) and 2,3-butanediol. Genes involved in motility and chemotaxis, along with those encoding exopolysaccharides (EPSs) and the biofilm matrix protein TasA, which are required for plant root colonization (9), were also identified. Furthermore, the *aiiA* gene encoding a lactonase that inactivates acyl-homoserine lactones involved in regulating virulence gene expression in many plant pathogens (10) was detected in strain KnB1. Collectively, these findings suggest that *B. subtilis* strain KnB1 has strong potential as a biocontrol agent for managing plant diseases.

## Data Availability

The whole-genome sequencing project has been deposited at NCBI under the BioProject accession number PRJNA1273501 and the Biosample accession number SAMN48948952. Raw sequences from Illumina and ONT sequencing have been deposited to the Sequencing Read Archive under the accession numbers SRR34330778 and SRR34330779, respectively. The sequenced genomic data have been deposited in GenBank under the accession number CP196553.

## References

[1] Arkin AP, Cottingham RW, Henry CS, Harris NL, Stevens RL, Maslov S, Dehal P, Ware D, Perez F, Canon S, et al.. 2018. KBase: The United States Department of Energy Systems Biology Knowledgebase. Nat Biotechnol 36:566–569. doi:10.1038/nbt.4163 29979655 PMC6870991

[2] Martin M. 2011. Cutadapt removes adapter sequences from high-throughput sequencing reads. EMBnet J 17:10. doi:10.14806/ej.17.1.200

[3] Wick RR. 2025. Filtlong. https://github.com/rrwick/Filtlong.

[4] Wick RR, Judd LM, Gorrie CL, Holt KE. 2017. Unicycler: resolving bacterial genome assemblies from short and long sequencing reads. PLoS Comput Biol 13:e1005595. doi:10.1371/journal.pcbi.1005595 28594827 PMC5481147

[5] Parks DH, Imelfort M, Skennerton CT, Hugenholtz P, Tyson GW. 2015. CheckM: assessing the quality of microbial genomes recovered from isolates, single cells, and metagenomes. Genome Res 25:1043–1055. doi:10.1101/gr.186072.114 25977477 PMC4484387

[6] Tatusova T, DiCuccio M, Badretdin A, Chetvernin V, Nawrocki EP, Zaslavsky L, Lomsadze A, Pruitt KD, Borodovsky M, Ostell J. 2016. NCBI prokaryotic genome annotation pipeline. Nucleic Acids Res 44:6614–6624. doi:10.1093/nar/gkw569 27342282 PMC5001611

[7] Blin K, Shaw S, Vader L, Szenei J, Reitz ZL, Augustijn HE, Cediel-Becerra JDD, de Crécy-Lagard V, Koetsier RA, Williams SE, et al.. 2025. antiSMASH 8.0: extended gene cluster detection capabilities and analyses of chemistry, enzymology, and regulation. Nucleic Acids Res 53:W32–W38. doi:10.1093/nar/gkaf334 40276974 PMC12230676

[8] Aziz RK, Bartels D, Best AA, DeJongh M, Disz T, Edwards RA, Formsma K, Gerdes S, Glass EM, Kubal M, et al.. 2008. The RAST Server: rapid annotations using subsystems technology. BMC Genomics 9:75. doi:10.1186/1471-2164-9-75 18261238 PMC2265698

[9] Beauregard PB, Chai Y, Vlamakis H, Losick R, Kolter R. 2013. Bacillus subtilis biofilm induction by plant polysaccharides. Proc Natl Acad Sci USA 110:E1621–30. doi:10.1073/pnas.1218984110 23569226 PMC3637697

[10] Dong YH, Xu JL, Li XZ, Zhang LH. 2000. AiiA, an enzyme that inactivates the acylhomoserine lactone quorum-sensing signal and attenuates the virulence of Erwinia carotovora. Proc Natl Acad Sci USA 97:3526–3531. doi:10.1073/pnas.97.7.3526 10716724 PMC16273

